# The state of evolutionary medicine in undergraduate education

**DOI:** 10.1093/emph/eoz012

**Published:** 2019-05-09

**Authors:** Daniel Z Grunspan, Karla T Moeller, Randolph M Nesse, Sara E Brownell

**Affiliations:** 1 Center for Evolution and Medicine, Arizona State University, 427 E. Tyler Mall, Tempe, AZ, USA; 2 School of Life Sciences, Arizona State University, 427 E. Tyler Mall, Tempe, AZ, USA

**Keywords:** undergraduate education, instruction, evolution and medicine, survey, education, undergraduate courses

## Abstract

**Background and objectives:**

Undergraduate courses that include evolutionary medicine (EM) are increasingly available, but quantified data about such courses are lacking. In this article, we describe relevant course offerings by institution and department type, in conjunction with information on the backgrounds and experiences of associated instructors.

**Methodology:**

We searched course catalogs from 196 American universities to find courses that include EM, and sent a survey to 101 EM instructors to ask about their backgrounds and teaching experiences.

**Results:**

Research-focused universities (R1) were much more likely to offer at least one course that covers evolutionary applications to health and disease than universities that granted only bachelor’s or master’s degrees. A survey course on EM was offered in 56% of 116 R1 universities, but only 2% of the 80 non-R1 universities we searched. Most EM instructors have backgrounds in anthropology or biology; each instructor’s area of expertise provides clues as to how continued growth of EM may occur differently by discipline.

**Conclusions and implications:**

Undergraduates are most likely to learn about EM in research-intensive universities from an anthropological or biological perspective. Responses from anthropology and biology instructors, including whom they share course materials with, highlight that courses may differ depending on the discipline in which they are taught.

**LAY SUMMARY:**

Recognition of evolution’s relevance to understanding health and disease is growing, but documentation of coverage in undergraduate education is lacking. This study explores where evolutionary medicine (EM) content is taught across 196 undergraduate institutions and how 53 instructors describe their experiences teaching EM.

## INTRODUCTION

Evolutionary medicine (EM) applies an evolutionary lens to medically relevant topics to deepen our understanding of disease and treatment strategies. The field’s great potential for improving human health has led to growing calls to include the principles of evolutionary biology in curricula for future health professionals [1–4]. Medical schools have been slow to answer the call with overcrowded curricula often cited as the reason [5, 6]. An alternative approach is to provide training in EM in undergraduate courses that will likely be taken by future medical professionals.

The integration of EM into the undergraduate curriculum can bolster the experiences of students in multiple ways. First, examples of human health and disease can improve undergraduate engagement with evolution by highlighting examples that may be personally relevant for many students [7], especially those interested in health careers. Second, EM examples can also be a way to include the core concepts of evolution in medically relevant undergraduate courses such as anatomy, biochemistry, physiology and microbiology [8, 9].

However, little is known about the extent to which EM is being taught at the undergraduate level, what types of institutions are offering EM courses, or the backgrounds and experiences of EM instructors. To date, the only published data on EM’s presence in undergraduate education come from a previous survey that found that about half of 32 collected syllabi from undergraduate evolution courses at large, public universities included some mention of EM [10]. Still missing, however, are data on other types of courses that include EM, the departments and universities that offer these courses, and the backgrounds of instructors that teach these courses. Collecting baseline knowledge about the current state of EM education would establish an important foundation for future educational initiatives. For example, pedagogical resources for teaching EM will be most helpful if they are developed while keeping in mind common struggles students face while learning EM. The prior teaching experiences of EM instructors are particularly helpful for understanding these student struggles. Instructor experiences are also an important indicator of how the field has grown in the past and may continue to grow in the future. For example, knowing how current instructors became a part of the larger EM community or came to teach EM provides important indicators of how the field may be growing, including how this growth may differ by discipline.

Previous work in EM education has suggested learning goals for medical students [1, 2] and premedical students [7], described principles of EM [11, 12] or EM classes [13], and has provided teaching examples for use in EM courses [9, 14]. A recent study used a Delphi method encompassing four surveys and enlisting the opinions of 56 international EM experts in an effort to identify 14 core principles of EM [Box 1] [15]. These core principles are important for students to understand because they tie together the vast amount of content that makes up EM. However, little is known about the extent to which instructors view these principles as important to the learning goals of undergraduate courses, and whether instructors from different backgrounds emphasize these principles differentially in their courses. The influence of instructor background on content may be particularly strong in EM courses relative to more established course subjects, such as anatomy and physiology or evolution and ecology.

Box 1. Core principles of EM

*Types of Explanation*: Both proximate (mechanistic) and ultimate (evolutionary) explanations are needed to provide a full biological understanding of traits, including those that increase vulnerability to disease.

*Evolutionary Processes*: All evolutionary processes, including natural selection, genetic drift, mutation, migration and non-random mating, are important for understanding traits and disease.

*Reproductive Success*: Natural selection maximizes reproductive success, sometimes at the expense of health and longevity.

*Sexual Selection*: Sexual selection shapes traits that result in different health risks between sexes.

*Constraints*: Several constraints inhibit the capacity of natural selection to shape traits that are hypothetically optimal for health.

*Trade-offs*: Evolutionary changes in one trait that improve fitness can be linked to changes in other traits that decrease fitness.

*LHT*: Life history traits, such as age at first reproduction, reproductive lifespan and rate of senescence, are shaped by evolution, and have implications for health and disease.

*Levels of Selection*: Vulnerabilities to disease can result when selection has opposing effects at different levels (e.g. genetic elements, cells, organisms, kin and other levels).

*Phylogeny*: Tracing phylogenetic relationships for species, populations, traits or pathogens can provide insights into health and disease.

*Coevolution*: Coevolution among species can influence health and disease (e.g. evolutionary arms races and mutualistic relationships such as those seen in the microbiome).

*Plasticity*: Environmental factors can shift developmental trajectories in ways that influence health and the plasticity of these trajectories can be the product of evolved adaptive mechanisms.

*Defenses*: Many signs and symptoms of disease (e.g. fever) are useful defenses, which can be pathological if dysregulated.

*Mismatch*: Disease risks can be altered for organisms living in environments that differ from those in which their ancestors evolved.

*Cultural Practices*: Cultural practices can influence the evolution of humans and other species (including pathogens), in ways that can affect health and disease (e.g. antibiotic use, birth practices and diet).

### Research objectives

Our first research objective is to report where and in what capacity EM is currently offered in undergraduate curriculum. In doing so, we provide a baseline measure of EM’s presence in undergraduate curricula that will enable tracking future growth. Our second research objective is to describe the background and experiences of instructors teaching EM courses, and how variations in instructor background influence teaching.

## METHODS

### Course catalog search

Two researchers (DZG and KTM) independently searched online course catalogs from an initial sample of 120 American universities for courses that include EM content. The selected course catalogs came from a random subset of 40 Baccalaureate Colleges with Arts & Sciences Focus (BCASFs), 40 master’s granting institutions and 40 research-intensive (R1) institutions, as identified by the Carnegie classification system [16]. Because courses may focus on EM with different degrees of specificity, course descriptions were used to identify courses of three general categories: courses that were entirely devoted to evolutionary applications to health and disease (Category 1), courses that applied evolution to specific health-related topics, such as the evolution of infectious disease (Category 2), and courses that included evolutionary applications to health and disease as part of a more general curriculum (Category 3). Course contents were only identifiable through course catalog descriptions and course titles, thus these methods may underestimate the number of courses that include EM content, particularly those that might fit Category 3. The two researchers compared their categorization of each relevant course and came to consensus on any disagreements regarding classification. The search criteria are included in the Supplementary Materials.

### Instructor survey

We were interested in the perspectives of instructors teaching courses entirely devoted to EM (Category 1). To increase the number of identified instructors teaching these types of courses, the same two researchers (DZG and KTM) searched for Category 1 courses at 76 additional R1 institutions that were not previously randomly selected (there are 116 total R1 universities). R1 institutions were chosen because, as described below, they were significantly more likely to offer courses surveying EM, and thus represented an efficient way to identify an initial list of EM instructors. Instructor names and contact information were recorded whenever this information was publicly available online.

The identified instructors were surveyed about their backgrounds and experiences. The survey assessed: (i) the academic and professional backgrounds of EM instructors; (ii) the instructors’ backgrounds with EM, including their involvement in research and how they first learned about EM; (iii) the learning goals of their courses, including the importance of EM core principles; (iv) difficulties their students have experienced learning EM; and (v) informal sharing networks of EM course materials. To identify additional instructors of EM courses, we also asked participants to list colleagues they know who teach EM courses. We sent this same survey to instructors that were identified by participants in this initial wave.

Seventy-nine instructors whose contact information was found online were initially sent the survey. Respondents were asked to list any colleagues who they knew were also teaching EM courses; respondents from this first wave listed 22 additional faculty that had not been previously identified. The survey was sent to these additional faculty in a second wave, resulting in a total of 101 instructors receiving the survey. Fifty-three instructors completed the survey for a response rate of 52.5%.

### Analyses

We were interested in whether the prevalence of EM courses differed by type of institution. We used Fisher’s exact test to examine whether the proportion of schools that offered any EM courses (Category 1, 2 or 3) differed between different university types (R1, BCASF and master’s granting).

Analyses of the instructor survey focused on differences in how EM was taught in biology versus anthropology departments. We used Fisher’s exact test to test whether the proportion of instructors who have ever been involved in EM research was the same between anthropologists and biologists. We used Mann–Whitney U tests with a Bonferroni correction for multiple comparisons to test whether anthropologists and biologists reported the same level of importance for each of the 14 core principles.

Network data were plotted using the *statnet* and *Ggally* packages in R [15, 17, 18]. We tested for a relationship between instructor degree centrality and the importance of the 14 core principles as rated by each instructor using a Spearman’s correlation test.

The local IRB review determined this research was exempt (STUDY00007313).

## RESULTS

### Over half of R1 universities offer a full course devoted to EM

At least one EM course of any type was available at 92.5% of R1 institutions, 40% of BCASFs and 22.5% of master’s granting universities (Table 1, Supplementary Table S1). R1 universities were significantly more likely to offer a course that teaches EM compared to BCASFs or master’s granting institutions.

**Table 1.** Frequency of BCASFs, Master's granting institutions and research-intensive institutions (R1) with different types of courses that teach evolutionary applications to health and disease

	Total schools examined	Number of schools with any EM class found	Number of schools with no EM class found	Number of schools with Category 1^a^ class(es)	Number of schools with Category 2^b^ class(es)	Number of schools with Category 3^c^ class(es)
BCASFs	40	16 (40%)	24 (60%)	1 (2.5%)	8 (20%)	12 (30%)
Master's granting	40	9 (22.5%)	31 (77.5%)	1 (2.5%)	2 (5%)	8 (20%)
R1	40	37 (92.5%)	3 (7.5%)	18 (45%)	24 (60%)	30 (75%)

a Category 1 refers to classes that are entirely focused on EM.

b Category 2 refers to classes that are focused on a specified application of evolution to a health topic, such as evolution of infectious diseases or evolution and mental disorders.

c Category 3 refers to classes that include some mention of applying evolution to health or disease, but only as one piece of a larger class.

### EM is primarily taught in biology and anthropology departments

Of the 181 identified EM courses across the 120 sampled universities (Table 2), most (134/181) were offered by biology or anthropology departments. A few were offered by a biomedical department (e.g. veterinary medicine, pathology and epidemiology), psychology, or by biomedical engineering departments. Some were cross-listed between two departments, most frequently between anthropology and biology departments.

**Table 2.** Number of courses found by type of course and department

	Number of Category 1^a^ courses	Number of Category 2^b^ courses	Number of Category 3^c^ courses	Total number of courses
Anthropology	6	11	17	34
Biology	14	27	59	100
Biomedical	1	5	3	9
Other	3	6	12	21
Cross-listed	5	7	5	17
Total	29	56	96	181

a Category 1 refers to classes that are entirely focused on EM.

b Category 2 refers to classes that are focused on a specified application of evolution to a health topic, such as evolution of infectious diseases or evolution and mental disorders.

c Category 3 refers to classes that include some mention of applying evolution to health or disease, but only as one piece of a larger class.

### Anthropology and biology faculty differ in their experiences with EM

Almost all EM instructors responding to the survey had academic backgrounds, as defined by their highest earned degree: 21 in anthropology, 24 in a biological field, six with medical degrees and two with degrees in other fields.

Instructors were asked how they first learned about EM. Common responses included taking a course as an undergraduate or graduate student, learning about the field because of developments in their own research, learning about EM directly from another person, and reading books or articles on EM. Anthropologists and biologists differed in the ways they learned about EM (Table 3). Anthropology faculty more frequently cited experiences as an undergraduate (7 out of 23 anthropologists compared to 2 out of 21 biologists) or as a graduate student (10 out of 23 anthropologists compared to 4 out of 21 biologists). However, biologists more commonly described their own research leading to their introduction to EM (9 out of 21 biologists mentioned research) compared to anthropologists (3 out of 23 anthropologists mentioned research). These are surprising findings in light of the fact that EM courses are more frequently offered through biology departments than anthropology departments (Table 2). This may reflect a generational difference in the presence of EM in curriculum between biology and anthropology.

**Table 3.** Ways instructors mentioned first learning about EM by disciplinary area of their highest degree

	Anthropology	Biology	MD	Other
Undergraduate	7 (30.4%)	1 (5.3%)	2 (50.0%)	0 (0%)
Graduate school	10 (43.5%)	3 (15.8%)	1 (25.0%)	0 (0%)
Post-doctoral experience	1 (4.3%)	2 (10.5%)	0 (0%)	1 (50.0%)
Class	8 (34.8%)	3 (15.8%)	1 (25.0%)	0 (0%)
Own research	3 (13.0%)	8 (42.1%)	3 (75.0%)	1 (50.0%)
Direct from a specific person	2 (8.7%)	7 (36.8%)	2 (50.0%)	0 (0%)
Reading about EM	9 (38.1%)	5 (26.3%)	1 (25.0%)	1 (50.0%)

Categories are not mutually exclusive. For example, first exposure through an undergraduate course counts under both ‘Undergraduate’ and ‘Class’.

Faculty were asked whether they are currently, have previously been, or were never involved in EM research. More biologists than anthropologists described research activities as responsible for their initial exposure to EM, but fewer biologists than anthropologists reported current or previous involvement with EM research. All four medical doctors and two instructors from other academic backgrounds reported being currently involved in EM research (Table 4).

**Table 4.** Survey respondents’ background with research in EM by their disciplinary background

	Anthropology (*n* = 22)	Biology (*n* = 25)	MD (*n* = 4)	Other (*n* = 2)
Currently involved in EM research	17 (77.3%)	7 (28.0%)	4 (100%)	2 (100%)
Never involved in EM research	3 (13.6%)	16 (64.0%)	0 (0%)	0 (0%)
Previously involved in EM research	2 (9.1%)	2 (8.0%)	0 (0%)	0 (0%)

### Faculty share teaching resources primarily within their own discipline

Undergraduate courses can be constructed *de novo* without the use of materials developed for other courses, or they may integrate previously developed materials. Faculty may share syllabi, reading lists, lecture slides, test questions or other classroom activities, which can lead to similarities between the content and teaching activities of different courses. Courses developed independently, without any material sharing, may be more likely to differ in content or teaching activities on average. Given that EM courses are offered across disciplines, we were interested in whether sharing networks of course materials reflect traditional academic disciplinary structures. We asked survey participants to list up to six individuals that they have either provided materials to, or received materials from, for their EM courses.

**Figure 1.**
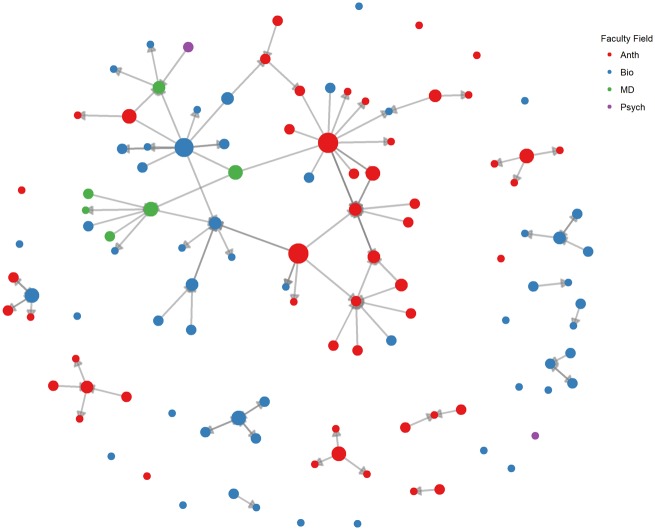
Material-sharing network among survey participants. Nodes represent instructors, with node size corresponding to the number of other instructors receiving course materials from the focal node. Arrows indicate direction of material sharing

Many instructors listed other survey participants as both recipients and donors of course materials, but other listed individuals were either not identified as teaching a Category 1 course through our methods (*n* = 48) or were sent the survey (*n* = 11), but did not complete it. The disciplines of these additional instructors, 59 in total, were available online through personal or academic webpages. Because we were interested in the spread of course materials within and between disciplines, we identified and attached data about the disciplinary foci of these individuals to the network data. Figure 1 displays a sociograph of this course-material-sharing network.

There are several takeaways from this network. First, a large proportion of EM instructors (36/53) share course materials. This materials-sharing community may work to reinforce curricula that are similar among these instructors’ courses. Second, faculty tend to exchange course materials with others from the same discipline. Third, the existence of many isolated nodes and smaller communities suggests that many instructors develop courses without using shared materials.

### Core principles tend to be important, but vary by course

Instructors evaluated the importance to their course of 14 previously described core principles of EM [12] from ‘not important’ to ‘essential’ for their students to learn. Generally, instructors rated all core principles as ideas important for students in their courses. However, 12 of the 14 core principles were rated as either ‘not important’ or ‘slightly important’ by at least one instructor, indicating that variation exists. Only two principles were rated as being at least moderately important for students to understand for all of the instructors’ courses: evolutionary processes and reproductive success. It is noteworthy that the three principles that instructors rated as least important were sexual selection, multiple levels of selection and phylogeny; these three principles were also the three lowest rated among the panel of experts that helped define the core principles [12].

**Figure 2.**
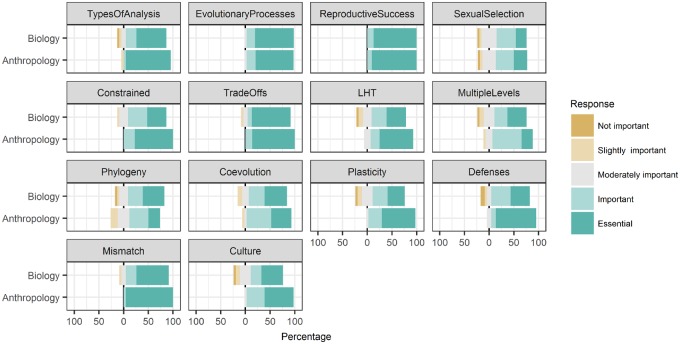
Instructor-reported importance of 14 core principles of EM to their courses, organized by instructor disciplinary background. Percentages from left to right indicate the percent of ‘Not important’ or ‘Slightly important’ responses, the percent of ‘Moderately important’ responses and the percent of ‘Important’ or ‘Essential’ responses, respectively

Anthropologists tended to rate core principles as more important to their courses than did biologists (Fig. 2). However, the only principle that was rated significantly different between biologists and anthropologists was evolved defenses, which was rated more important in courses taught by anthropologists. It is worth noting that 78% of biologists still rated evolved defenses as either important or essential to their course.

### Students in all courses faced common struggles

We asked instructors to identify any struggles that students had with content in their EM courses. The most common theme was that students had difficulty understanding general evolutionary concepts, such as genetic drift or macroevolution (28 out of 48 responses). The second most common theme was the influence of student backgrounds, with instructors qualifying their responses by mentioning how the disciplinary background or lack of student prerequisite knowledge impacted their courses (12 out of 48 responses). For example, instructors reported anthropology students having difficulty with biological content such as immunology, genomics and basic evolution, or biology students having difficulty with topics such as paleoanthropology or integrating and discussing the importance of culture. Some instructors reported ‘non-majors’ lacking prerequisite knowledge. These responses reflect the difficulty of teaching a course that focuses on evolution, integrates a breadth of disciplines and often enrolls groups of students that have differing prior knowledge. A full list of responses to this question is available in the Supplementary Materials (Supplementary Table S2).

## DISCUSSION

Courses that teach EM at the undergraduate level are an important component for making evolution a basic science for medicine. Our study summarizes information on the prevalence of such courses across different institution types, who teaches EM courses, what principles are valued by these instructors, and common barriers that impede student learning.

### EM is common at R1 institutions, but is mostly absent from other institutions

It is, perhaps, not surprising that research-intensive universities offer more courses that include evolutionary applications to health and disease. These institutions are larger (Supplementary Table S1 and Fig. S3) and can offer more courses on specialized topics at the undergraduate level. It is unclear from our data whether the intensity of EM research at an institution is associated with a higher likelihood of offering EM courses. However, our data suggest that professors who are actively engaged in EM research, or whose own research led them to learning about EM, are the ones developing EM courses. Thus, it may be that the personnel at less research-intensive universities are either unaware of EM or lack necessary resources to develop EM courses at their university.

In relation to these findings, undergraduates have more opportunities to learn about EM if they attend a research-intensive university. Increasing the presence of EM across more diverse institutions can help reach more students, including many who will pursue health professions. Understanding the barriers to teaching EM can help increase offerings in non-R1 settings and can inform the types of resources that would facilitate the adoption of EM into a broader range of courses.

### Expanding the field of EM through education

Increasing the exposure of pre-med students to EM may be best accomplished by finding ways to integrate EM into introductory biology and upper level evolution courses, which provide various opportunities to integrate relevant health examples. Indeed, several commonly used evolution textbooks already include sections on EM or human health [19–22].

Knowledge about the backgrounds of current instructors can inform how EM may continue to grow through specialized courses that focus specifically on evolution and medicine. Information on how current instructors were initially exposed to EM and their current involvement with EM research highlights discipline-specific patterns for how faculty came to teach EM. Biologists teaching EM were more likely to learn about EM outside of formal educational training, commonly through tangential ties to their own research or by meeting someone already involved in EM. Anthropologists teaching EM were more likely to gain exposure through more formal educational routes, such as past coursework. Given the growth of EM thus far, in the form of an international society, a journal, and the prevalence of courses at research-intensive universities, it is likely that the opportunities for faculty and students to discover EM are greater than ever. Members of the EM community, including current instructors, are central to this growth opportunity. While expanding the presence of EM in classrooms and curricula can take formal routes, such as the creation of national EM centers, personal connections—including those with former teachers, mentors and colleagues—were listed as memorable routes of initial exposure to EM. Individuals invested in EM can inspire similar passions in current and future undergraduates, mentees and peers to continue the growth of an engaged EM community. Creating effective undergraduate EM courses is a major part of this opportunity.

### Approaches to consider for teaching EM at the undergrad level

It was not clear from our survey how similar or different EM courses were between disciplines or instructors. However, as EM continues to grow and take shape as a field, consideration of the advantages and disadvantages of a consistent approach to undergraduate courses would be worthwhile. Conversations and resources regarding what to teach in EM courses have centered on what medical professionals should know [2–6, 23]. Most undergraduate EM courses are offered within a single discipline, which raises important questions for EM undergraduate education; how should EM learning goals integrate across disciplines, and how similar or different should EM curriculum be across the different disciplines that offer EM courses?

Instructors indicated similar importance of core principles for EM courses, regardless of their discipline. By definition, core principles have broad applications [24] and an interdisciplinary panel reached consensus agreement about their importance, so this is not entirely surprising. However, the contexts in which these principles are applied in EM courses may differ by discipline in ways that our survey could not capture. For example, the principle of trade-offs can be exemplified through molecular examples, ecological examples or life-history examples. Instructors may place greater importance on certain applications of these principles depending on the discipline in which the course is being taught. EM courses across different disciplines may also differ in their learning objectives. Biology and anthropology EM courses may disproportionately focus on different aspects of evolution’s relevance that align with their own department’s curricular goals; biology courses may focus mostly on molecular evolution or co-evolutionary dynamics of hosts and pathogens, while anthropology courses may emphasize cultural evolutionary histories of human proximity to animals. All of these approaches use evolutionary thinking and tools, and all have applications for health and disease, but each approach may be more appropriate for students in either anthropology or biology. We encourage future research exploring whether this may be true.

The cross-disciplinary nature of EM creates a question for those developing educational resources in EM—can resources be one size fits all, or do disciplinary differences mean that a lesson relevant for one instructor may not meet the curricular needs of the next? A potential alternative solution is to focus efforts on leveraging EM’s multi-disciplinary nature to teach students to think across disciplinary boundaries. EM offers suitable subject matter for combining multiple disciplinary perspectives, which may consider EM to be either interdisciplinary or multidisciplinary. Interdisciplinarity has been defined as an approach that combines different disciplines to develop new methods, concepts or other products that are required to solve a question that cannot be answered through a single disciplinary approach [25]. Multidisciplinarity draws on multiple viewpoints to offer new solutions to old problems [26]. A precise measure of the extent to which this is already occurring is not clear from our data, but patterns from the materials-sharing network, the departmental specificity of courses, and the lack of any explicit discussion of interdisciplinary learning goals suggest that EM as a means for teaching interdisciplinary or multidisciplinary skills may be uncommon. However, many instructors mentioned critical thinking as an important learning goal, which is often included as an important outcome for interdisciplinary courses [27].

Many major problems faced by the world, including those in medicine and public health, are not mono-disciplinary in nature, leading to calls for more and improved interdisciplinary curricula [9, 28]. EM expands medical and public health fields by highlighting the role of evolutionary perspectives, adding an extra disciplinary layer to the already transdisciplinary area of health and medicine. In doing so, EM further brings together evolutionary biology, anthropology, physiology, molecular and developmental biology and other fields such as psychology. For example, students tasked with understanding the rising rates of obesity can consider evolved reward pathways, social disparities, genetic variation and mismatches present in modern environments, and how these may work together to untangle a complex and important health problem. EM may represent an area conducive to breaking disciplinary barriers for students, and developing pedagogical approaches that explicitly focus on interdisciplinary training in EM may be a promising direction. Developing educational resources for EM that provide opportunities for students to develop interdisciplinary skills would not only benefit students in EM, but may be attractive to departments, including those in less research-intensive schools, to include EM in their curriculum.

### Common struggles highlight educational challenges in EM

Common difficulties from the perspectives of instructors in EM courses represent important problems to solve, and may be deterrents to the growth of the field. A commonly mentioned issue by EM instructors was the breadth of prerequisite knowledge required of students to be able to engage with EM. Instructors often pointed out specific deficiencies that students with anthropology or biology backgrounds faced that made teaching the course particularly difficult. In designing courses that aim to integrate these fields, faculty must consider that biology students may have weak backgrounds in paleoanthropology and integrating culture into understanding evolution, while anthropology students may have weaker foundations in subjects such as molecular and evolutionary biology. Overcoming these issues represents a special challenge that must be considered in course design. Ensuring appropriate prerequisite courses, co-developing courses with interdisciplinary teams, or making interdisciplinary scholarship an explicit part of course design while leveraging the unique disciplinary backgrounds of students [29] may be productive ways to teach EM in an interdisciplinary way that supports diverse student backgrounds and expertise.

Understanding evolution was the most common student difficulty mentioned by instructors. While evolution is in many ways an eloquent process, it includes many concepts that are notoriously difficult for students to understand and students often harbor misconceptions [30–32]. Furthermore, student understanding of evolution is prone to misunderstandings stemming from cognitive heuristics [33–35]. Many students enter college without having been exposed to the mechanics of evolution, as many high school teachers either entirely avoid or only cursorily teach evolution [36]. Many students also struggle reconciling their religious identity with evolution [37], presenting a potential source of conflict. However, at all levels of learning, EM provides a unique context for teaching evolution that most students can relate to on a personal level. Understanding how this context improves student engagement with evolution and health would highlight the benefits of integrating EM into a variety of courses across K-16. Research on the benefits of EM in high school is growing [38], and more resources are becoming available for teachers at high school and higher grade levels to use EM as a way to introduce evolution [39]. Encouraging or contributing to EM integration across grade levels may help address this issue in the future.

Understanding how to address student misconceptions about evolution with EM courses first requires considering how learning goals are defined in different EM courses. For example, evolution may be a ‘threshold concept’ that is a necessary prerequisite for students to learn EM. Threshold concepts are central ideas in a discipline that, once understood, help a learner ‘cross through a portal’ toward understanding [40]. Grasping a threshold concept is an irreversible experience that results in major conceptual shifts in understanding. Conceptualizing EM in this way suggests that evolution itself is not a core concept of EM, but instead a conceptual barrier to understanding other concepts that make up EM. Under this model, EM may be seen as an advanced topic best taught to students who are already experts in evolution and are not at risk of struggling due to their alternative conceptions of evolutionary processes [41]. However, this approach ignores the benefits of contextualized learning that EM offers. Alternatively, understanding evolution may be a core learning objective of an EM course. Indeed, evolution was frequently listed by instructors as a learning goal in their course, and is considered a core concept in biology and a core principle of EM [8, 9, 12]. Evolution is not typically seen as a threshold concept for biology [42]. Instead, there are threshold concepts that stand in the way of students understanding evolution, including ideas such as randomness, probability, temporal scale and spatial scale [43]. This conceptualization of EM suggests that it is a topic with a main goal of teaching evolution. In this case, student struggles with understanding evolution should not be considered as a barrier to learning, but instead are part of the learning progression.

### Study limitations

This work is meant to provide a broad view of the EM educational landscape, and as such, has several limitations. First, the scope of the course catalog survey was limited. Our sample was small relative to the large number of universities that exist. Indeed, more precise estimates would have been achieved with the inclusion of more institutions, on an international scale. However, for the purposes of gaining a general understanding of the state of EM education, our estimate is of value. Furthermore, a comparison to the only other previously published estimate of EM’s inclusion in courses, which had a similar sample size, corroborates our estimates. Our estimate of the frequency of schools offering courses that include a section on EM (Category 3 courses: 41.7% of the 120 schools) is similar to a previous study that found 50% of 32 evolutionary biology syllabi collected from 27 different institutions in the USA included EM in some way [10].

Our estimates are likely to under-represent the actual number of courses because they relied on course descriptions indicating that evolutionary applications to health and disease were a part of the course. It is likely that other courses include EM content but do not make it clear in these descriptions. However, at the same time, it is possible that course descriptions over-represent the presence of EM content, or that courses exist in the course catalog that are not regularly offered. Lastly, our data from instructors were not detailed enough to test finer-grain similarities or differences in course structure and content. A more thorough collection of class materials would allow a greater understanding of how disciplinary background of instructors impacts course design.

## CONCLUSION

We present a snapshot of the current landscape of EM in undergraduate education. Moving the needle toward greater representation of evolutionary applications to health and disease in undergraduate courses can take place in two main ways. First, more EM courses can be constructed. A second, non-mutually exclusive strategy is to push for greater inclusion of EM in courses that focus on human health and medicine, while courses focused on evolution can be infused with more medical and human examples in the form of EM [44]. Given the lack of representation of courses devoted to EM, especially at non-research-intensive universities, this strategy may be the lower hanging fruit. Indeed, it is likely easier to tweak current curriculum than to create curriculum anew.

## Supplementary Material

eoz012_Supplementary_Data
