# Supplementary material for: The state of evolutionary medicine in undergraduate education
Source: Evol Med Public Health. 2019 May 9;2019(1):82–92. doi: 10.1093/emph/eoz012 (PMC6557193; doi:10.1093/emph/eoz012)
Supplement: eoz012_Supplementary_Data [file eoz012_supplementary_data.zip › InstructorSurvey.pdf]

Default Question Block

We are interested in the background of individuals who teach courses on evolutionary medicine. The first section of this survey focuses on questions about your academic background, as well as your exposure to and experience with evolutionary medicine.

Which degrees have you completed?

- [ ] BA/BS
- [ ] MA/MS
- [ ] PhD
- [ ] MD
- [ ] DVM
- [ ] Other

Please answer the following questions with regards to your BA/BS.

In what field is your BA/BS?

From which college or university did you earn your BA/BS?

Please answer the following question with regards to your MA/MS.

In what field is your MA/MS?

From which college or university did you earn your MA/MS?

Please answer the following question with regards to your PhD.

In what field is your PhD?

From which college or university did you earn your PhD?

Please answer the following question with regards to your MD.

From which college or university did you earn your MD?

Please answer the following question with regards to your DVM.

From which college or university did you earn your DVM?

How did you first learn about the field of evolutionary medicine and become interested in it? Please describe specifically where, when, and how you first learned about it.

Please describe how you ended up teaching a course on evolutionary medicine.

What best describes the types of Evolutionary Medicine courses you have taught? Select all that apply.

- ☐ Full term undergraduate course focused on Evolutionary Medicine
- ☐ Full term graduate course focused on Evolutionary Medicine
- ☐ Undergraduate course not focused on Evolutionary Medicine, but with one or more class periods dedicated to evolutionary medicine
- ☐ Graduate course not focused on Evolutionary Medicine, but with a one or more class periods dedicated to it

☐ Other (describe below)

What best describes your involvement in Evolutionary Medicine research?

○ Currently conducting Evolutionary Medicine research
○ Not currently conducting Evolutionary Medicine research, but have been previously
○ Never have conducted Evolutionary Medicine research

We recognize that you may have taught many different courses that cover evolutionary medicine topics. The next section of this survey contains questions about courses *entirely focused on topics in evolutionary medicine*. Have you ever been involved in teaching a course that entirely focuses on topics in evolutionary medicine?

○ Yes
○ No

In this section, we are specifically interested in your most recent course that is *entirely focused on topics in evolutionary medicine*. Please answer the following questions with this course in mind.

What is the name and level of this course? (Example: Anthropology 250: Evolutionary Medicine).

In what institution and department did you most recently teach this Evolutionary Medicine course?

When was the last time you taught this course?

○ Within the past year
○ Between 1 to 3 years ago

- ◯ Longer than 3 years ago

How many times have you taught this class?

[ ]

What best describes the level of student this course is intended for? Select all that apply.

- ☐ Intro level undergraduate
- ☐ Upper level undergraduate
- ☐ Graduate level
- ☐ Medical or Veterinary students
- ☐ Health professionals

How does this course integrate into the department's degree structure?

- ◯ Required course as part of departmental degree
- ◯ Elective course as part of departmental degree
- ◯ Other [ ]

What text or other reading materials do you assign to students in your current Evolutionary Medicine course? Select all that apply.

- ☐ Primary scientific articles (e.g. peer reviewed articles in scientific journals)
- ☐ Popular science articles (e.g. news articles in outlets such as the New York Times or The Atlantic)
- ☐ *Body by Darwin* - Taylor
- ☐ *Evolution and Medicine* - Perlman
- ☐ *Evolution in Health and Disease* - Koella and Stearns
- ☐ *Evolutionary Medicine* - Medzhitov and Stearns
- ☐ *Evolutionary Medicine* - Trevathan and Smith
- ☐ *Evolutionary Medicine and Health: New Perspectives* - Smith and McKenna
- ☐ *Principles of Evolutionary Medicine* - Beedle, Hanson, and Gluckman
- ☐ *The Story of the Human Body: Evolution, Health and Disease* - Lieberman
- ☐ *Why We Get Sick* - Williams and Nesse
- ☐ *Zoobiquity* - Natterson-Horowitz and Bowers

☐ Other - describe below

What are your most important course goals for students in this Evolutionary Medicine course?

Please rate the extent to which each idea here is important for your students to understand in this Evolutionary Medicine class.

	Not important	Slightly important	Moderately important	Important	Essential
Explanations for biological traits range from proximate to ultimate.	○	○	○	○	○
Mechanisms of evolutionary processes (e.g. natural selection, mutation, drift, etc.).	○	○	○	○	○
Natural selection works to maximize reproductive success, not necessarily health or longevity.	○	○	○	○	○
Sexual selection and selection pressures that differ between males and females lead to different health risks between males and females.	○	○	○	○	○
Natural selection faces constraints (e.g. path dependence).	○	○	○	○	○
Evolutionary trade-offs.	○	○	○	○	○
Life History Theory.	○	○	○	○	○

	Not important	Slightly important	Moderately important	Important	Essential
Selection at levels other than individuals within populations (e.g. somatic selection of cells within individuals).	○	○	○	○	○
Phylogenetics and tree thinking.	○	○	○	○	○
Coevolution.	○	○	○	○	○
Phenotypic plasticity.	○	○	○	○	○
Evolved defenses (e.g. fever).	○	○	○	○	○
Evolutionary mismatch.	○	○	○	○	○
Culture and cultural influences on health.	○	○	○	○	○

In this section we are curious about your experiences teaching Evolutionary Medicine more generally. Please answer the following questions given all of your experiences teaching Evolutionary Medicine.

Have you faced any major challenges when creating a class in Evolutionary Medicine or getting it approved? If yes, please describe them below.

Have you faced any major challenges from students when teaching a class on Evolutionary Medicine? If yes, please describe them below.

Are there any concepts you perceive students in your class commonly struggle with when learning about Evolutionary Medicine? If yes, please describe them below.

We are interested in how collegial connections and shared materials between colleagues may impact the content of different evolutionary medicine courses. In this section, we are interested in the individuals from whom you have received or given classroom materials for evolutionary medicine courses.

Have you used any materials for your Evolutionary Medicine courses that came from colleagues or other individuals (e.g., syllabus, reading list, lecture slides, activities, test questions, etc.)?

- ○ Yes
- ○ No

Have you given any materials from one of your Evolutionary Medicine courses to colleagues or other individuals (e.g., syllabus, reading list, lecture slides, activities, test questions, etc.)?

- ○ Yes
- ○ No

Enter up to 6 people from whom have you *received* materials. Please enter full names if possible.

Name of Person 1	
Name of Person 2	
Name of Person 3	
Name of Person 4	
Name of Person 5	
Name of Person 6	

Enter up to 6 people to whom you have *sent* materials. Please enter full names if possible.

Name of Person 1	
Name of Person 2	
Name of Person 3	
Name of Person 4	
Name of Person 5	
Name of Person 6	

For each person below, please select the types of materials you *received from each individual listed*. Select all that apply.

	Syllabus	Reading list	Lecture slides	Class activitie
${q://QID32/ChoiceTextEntryValue/1}	☐	☐	☐	☐
${q://QID32/ChoiceTextEntryValue/2}	☐	☐	☐	☐
${q://QID32/ChoiceTextEntryValue/3}	☐	☐	☐	☐
${q://QID32/ChoiceTextEntryValue/4}	☐	☐	☐	☐
${q://QID32/ChoiceTextEntryValue/5}	☐	☐	☐	☐
${q://QID32/ChoiceTextEntryValue/6}	☐	☐	☐	☐

For each person below, please select the type of materials you *provided*.

	Syllabus	Reading list	Lecture slides	Class activitie
${q://QID37/ChoiceTextEntryValue/1}	☐	☐	☐	☐
${q://QID37/ChoiceTextEntryValue/2}	☐	☐	☐	☐
${q://QID37/ChoiceTextEntryValue/3}	☐	☐	☐	☐
${q://QID37/ChoiceTextEntryValue/4}	☐	☐	☐	☐
${q://QID37/ChoiceTextEntryValue/5}	☐	☐	☐	☐
${q://QID37/ChoiceTextEntryValue/6}	☐	☐	☐	☐

We are interested in surveying the community of individuals teaching evolutionary medicine in as much breadth as possible. Do you know of any colleagues currently teaching evolutionary medicine? If yes, could you please supply us with their contact information so we may forward them this survey?

Instructor name and e-mail

Instructor name and e-mail

Instructor name and e-mail

Instructor name and e-mail

Instructor name and e-mail

Instructor name and e-mail

Instructor name and e-mail

We are currently building a repository of teaching materials for individuals interested in or currently teaching evolutionary medicine. Would you be interested in being included in this materials network, and would you be willing to contribute any resources from your class (e.g. syllabus, lecture slides, activities, test questions, etc.)? If you respond yes, we will follow up with you with more information in the future.

- Yes, I'd like to access the repository, and would like to help contribute materials
- Yes, I'd like to access the repository when it is ready, but do not have materials to contribute
- No

We are interested in interviewing individuals who are teaching evolutionary medicine courses. Would you be interested in being part of a short interview sometime in the future about your experiences teaching evolutionary medicine?

- Yes
- No

Powered by Qualtrics
