# Supplementary material for: The state of evolutionary medicine in undergraduate education
Source: Evol Med Public Health. 2019 May 9;2019(1):82–92. doi: 10.1093/emph/eoz012 (PMC6557193; doi:10.1093/emph/eoz012)
Supplement: eoz012_Supplementary_Data [file eoz012_supplementary_data.zip › SupplementalMaterials4.19.19.docx]

**Search criteria for courses:**

1. Visit website of university from provided list and locate the university course catalog. For universities that have separate undergraduate and graduate course catalogs, complete steps 2 – 5 for each catalog. If catalogs are separated by year, complete steps 2 – 5 for the fall 2016, winter 2016, and spring 2017 catalog.
2. Search either the catalog as a whole, or the departments of natural sciences, life sciences, biology, anthropology, public health, or other related departments that would teach biology or health-focused courses. Once at a course list (preferably with course descriptions), use the control F feature to search for the term “evolution”.
3. Assess each mention of “evolution” to see if it is part of a course focusing on the use of evolutionary ideas in medicine and health.

- Category 1 courses are called evolutionary medicine or evolution and medicine, or something similar, such as human health in evolutionary perspective. The entire focus of the course is evolutionary medicine and it will include examples from various areas of medicine or public health.
- Category 2 courses have an evolutionary medicine theme, but focus on more specific content, such as evolution and mental disorders, or evolution of infectious disease, for example.
- Category 3 courses include some mention of evolution as it relates to medicine, health, or disease in the description of the course, as a focus, but this focus is does not make up the entirety of the class.

1. If a course appears to be a course in evolutionary medicine, record course information (name, course number, number of units). If an instructor is listed, record name; if last date course was taught is listed, record that as well.
2. Repeat your search in each course list for the terms “medicine” and “health”.
3. If you do not find qualifying courses, return to the university’s home page and search “evolution, medicine” in the university search bar. Investigate the first page (up to 6) results to search for a course in evolutionary medicine.
4. If you do not find qualifying courses, complete a Google search with the name of the university and the terms “evolution medicine”. Investigate the first page (up to 6) results to search for a course in evolutionary medicine at that university.
5. Enter all data (positive or negative) into provided EvMed Survey spreadsheet.
6. Restart process for the next university on the list.

Supplemental Table S1.

Frequency of Baccalaureate Colleges with Arts & Sciences Focus (BCASFs), master’s granting institutions, and research-intensive institutions (R1) with different types of courses that teach evolutionary applications to health and disease, broken down by size of universities as defined by Carnegie classifications.

|  | Size | Total schools examined | Number of schools with any EM class found | Number of schools with no EM class found | Number of schools with Category 1^a^ class(es) | Number of schools with Category 2^b^ class(es) | Number of schools with Category 3^c^ class(es) |
| --- | --- | --- | --- | --- | --- | --- | --- |
| BCASFs | Very Small | 12 | 1 | 11 | 0 | 0 | 1 |
| BCASFs | Small | 24 | 13 | 11 | 1 | 7 | 10 |
| BCASFs | Medium | 4 | 1 | 3 | 0 | 0 | 3 |
| BCASFs | Large | 0 | 0 | 0 | 0 | 0 | 0 |
| **BCASFs** | **TOTAL** | **40** | **15 (37.5%)** | **25 (62.5%)** | **1 (2.5%)** | **7 (17.5%)** | **14 (35%)** |
| Master’s granting | Very Small | 1 | 0 | 1 | 0 | 0 | 0 |
| Master’s granting | Small | 18 | 3 | 15 | 0 | 1 | 2 |
| Master’s granting | Medium | 18 | 6 | 12 | 1 | 1 | 6 |
| Master’s granting | Large | 3 | 0 | 3 | 0 | 0 | 0 |
| **Master’s granting** | **TOTAL** | **40** | **9 (22.5%)** | **31 (77.5%)** | **1 (2.5%)** | **2 (5%)** | **8 (20%)** |
| R1 | Very Small | 0 | 0 | 0 | 0 | 0 | 0 |
| R1 | Small | 1 | 1 | 0 | 0 | 0 | 1 |
| R1 | Medium | 3 | 3 | 0 | 1 | 1 | 2 |
| R1 | Large | 36 | 34 | 2 | 17 | 23 | 28 |
| **R1** | **TOTAL** | **40** | **38 (95%)** | **2 (5%)** | **18 (45%)** | **24 (60%)** | **31 (77.5%)** |
| ^a^Category 1 refers to classes that are entirely focused on evolutionary medicine.  ^b^Category 2 refers to classes that are focused on a specified application of evolution to a health topic, such as evolution of infectious diseases or evolution and mental disorders.  ^c^Category 3 refers to classes that include some mention of applying evolution to health or disease, but only as one piece of a larger class. | | | | | | | |

Figure S1: Frequencies in the number of EM courses of any type offered by institution type. Many R1 universities offer more than one course that covers EM, while many master’s granting and bachelor’s granting institutions offer none.


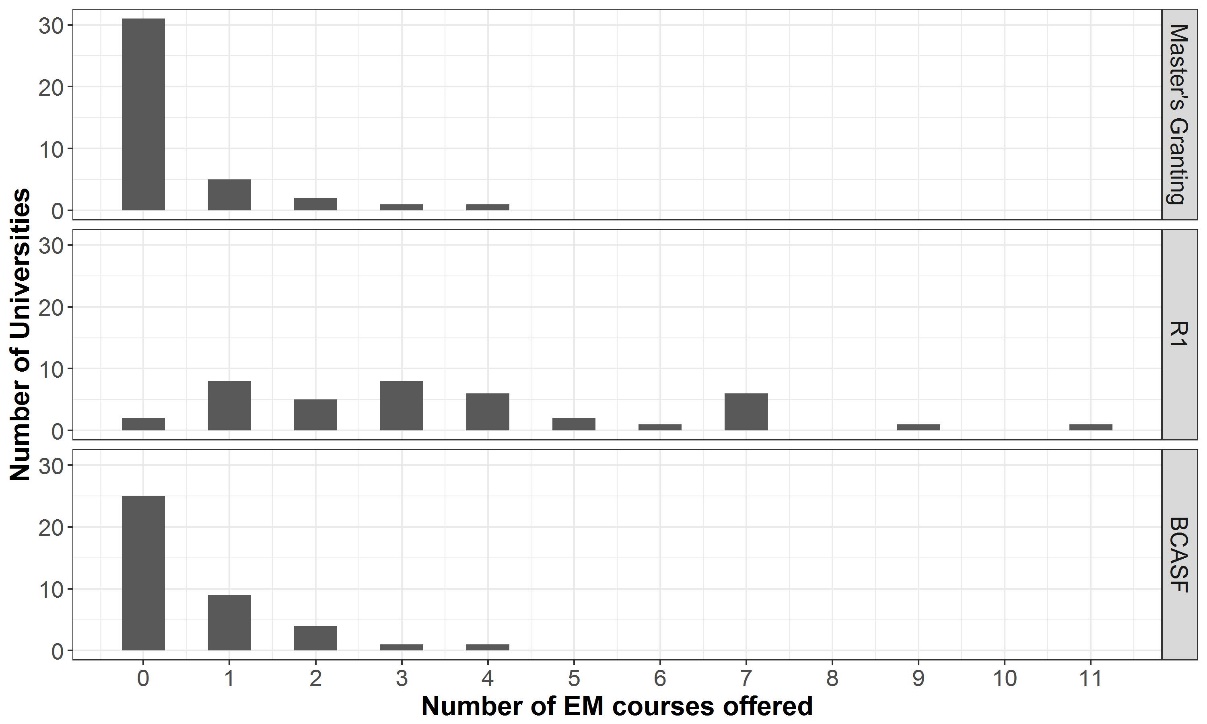


Figure S2: Frequencies in the number of courses surveying EM offered by institution type. Many R1 universities offer more than one course that covers EM, while many master’s granting and bachelor’s granting institutions offer none.


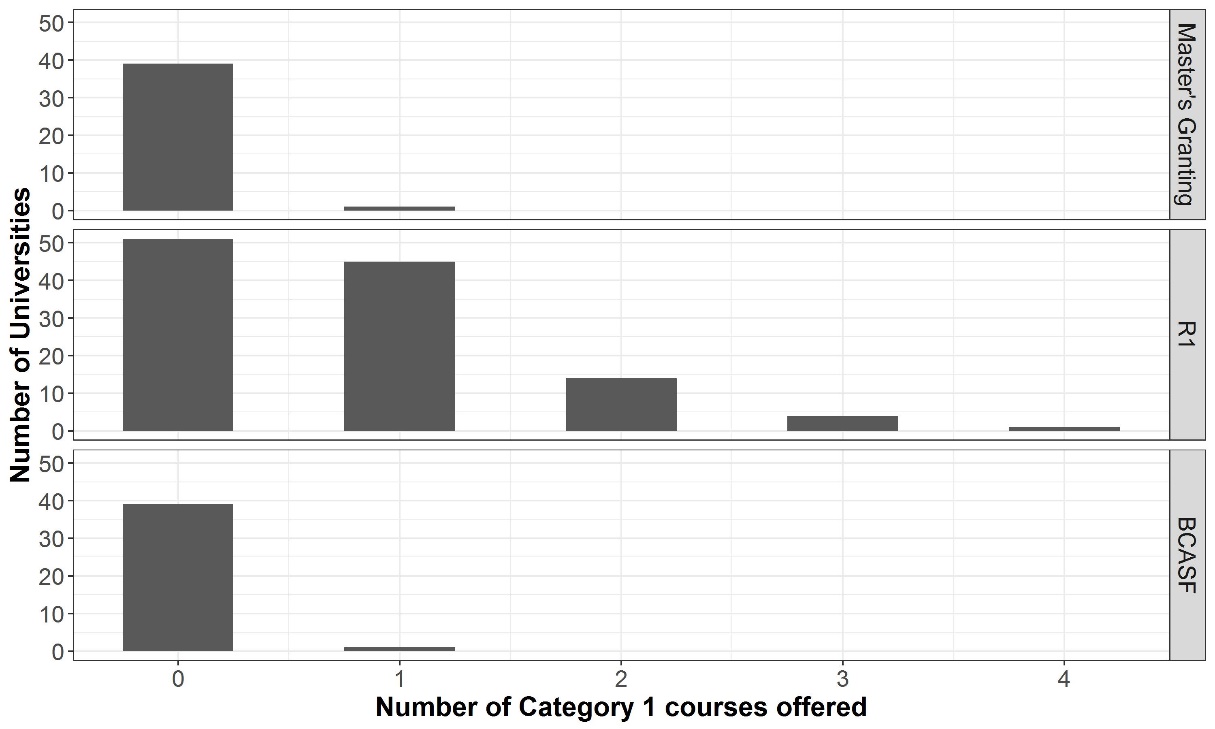


Figure S3: Heatmap of the number of total schools that are classified as master’s granting, R1, or Baccalaureate Colleges: Arts & Sciences by their size according to Carnegie classifications. Percentages are calculated as within each type of institution (master’s, R1, or BCASF).


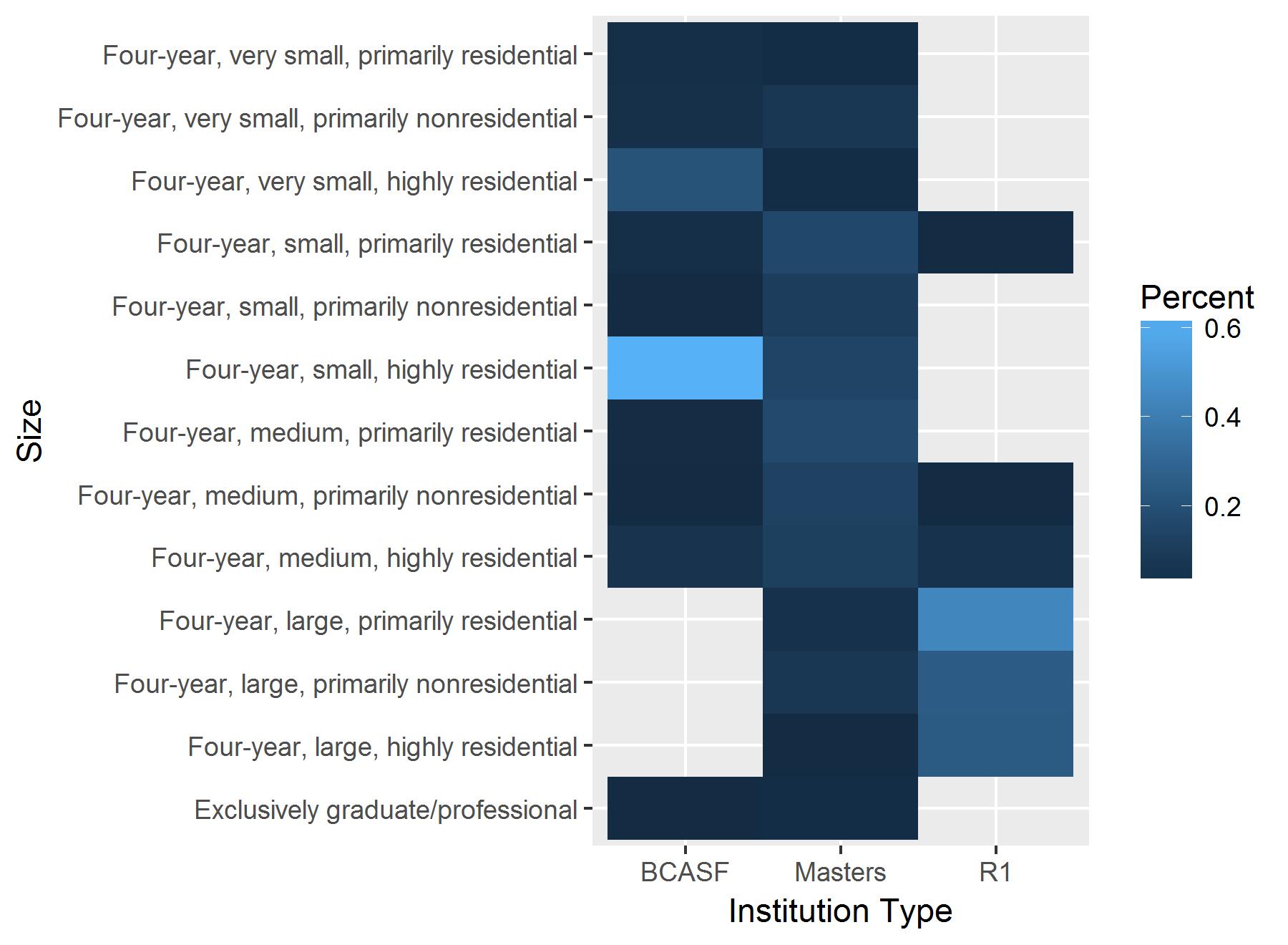


Supplemental Table S2: Responses to the question *“Are there any concepts you perceive students in your class commonly struggle with when learning about Evolutionary Medicine? If yes, please describe them below.”* Statements are included word for word.

| **Response** | **Mentions student background** | **Difficulty with Evolution mentioned** | **Nothing specific** | **Other** |
| --- | --- | --- | --- | --- |
| *A couple of the students struggled with the meaning of selected-effect function.* |  | ***X*** |  | ***X*** |
| *All of the genomics background is a lot to swallow. The course, everywhere I have taught it, is almost always offered as a science for non-science majors. Just to get to the point where we can talk about genomic regulation of traits requires some serious effort.* | ***X*** |  |  |  |
| *Basic evolution is always a challenge. Quantitative genetics confuses them and how complex traits evolve.* |  | ***X*** |  |  |
| *Basic evolution.* |  | ***X*** |  |  |
| *Depends on their background. If from a less biological background (and some are) they might struggle with various concepts and readings.* | ***X*** |  |  |  |
| *For anthropology students, actual evolutionary dynamics are hard, e.g. anything building off of understanding of population genetics or polymorphic traits. Plus you have to assume they have no working understanding of immunology.* | ***X*** | ***X*** |  |  |
| *Genetic drift is difficult for students in all of my courses. I have tried explaining it 50 different ways.* |  | ***X*** |  | ***X*** |
| *Genetics* |  |  |  |  |
| *I think some students still struggle with the proximate/ultimate dichotomy, and some continue to focus on proximate mechanisms to excess.* |  |  |  | ***X*** |
| *Initially they don't understand evolution by natural selection. Many have trouble understanding proximate and ultimate causes of many diseases.* |  | ***X*** |  |  |
| *It takes a while for them to really internalize the difference between fitness and health; the understanding that evolution is an on-going process not just something that happened in the past.* |  | ***X*** |  |  |
| *Like all students learning about evolution, students struggle a bit with understanding at which level selection acts and produces adaptations (cell, individual, group, etc.). Everyone gets mismatch and trade-offs.* |  | ***X*** |  |  |
| *Macroevolution and speciation.* |  | ***X*** |  |  |
| *Mismatch, trade-offs.* |  | ***X*** |  | ***X*** |
| *Mostly the students are stronger in anthropology, so understanding the speed of pathogen evolution, or frequency of horizontal gene transfer amongst microbes is a challenge.* | ***X*** | ***X*** |  | ***X*** |
| *My class, by necessity, does not have any formal prerequisites. This means that I teach students with a wide range of backgrounds. I also have both anth and biol majors (or intended majors) in the class. Some of the anth majors struggle with details of biology. Several of the biology majors who do not devote enough time to the class did not do well with what I considered to be an easy overview of paleoanthropology (e.g., in Lieberman's book). Most if not all find Stearns and Medzhitov challenging. I expected this would be the case, but in my view the main points are clear in that text. No one concept stands out as especially troublesome.* | ***X*** |  |  |  |
| *Natural selection, genetic drift, the various levels and interpretations of trade-offs and the mechanisms that cause them, the weaknesses in group selection arguments, thinking about variation in and among populations.* |  | ***X*** |  |  |
| *Population genetics (selective sweeps, etc.).* |  | ***X*** |  |  |
| *Population thinking is always hard, but this is also true of senior biology majors I have taught.* |  | ***X*** |  | ***X*** |
| *Quantitative issues, but not concepts.* |  |  |  |  |
| *Senescence is always tricky to get across, i.e. Medawar's mutation accumulation, Williams' antagonistic pleiotropy. I think the tricky part is that our society in general and most of our majors dislike mathematics, and both hypotheses are easiest to grok with numerical examples, e.g. looking at the effects on Ro of reducing fecundity by one offspring at different age classes, or by increasing current fecundity by 10% traded off against reducing survival to the next breeding episode by 10%.* |  | ***X*** |  | ***X*** |
| *Students always want black/white answers, and my goal is to teach them that "randomness" is the most important part of our life. And, also that adaptation does not underlie all traits and dispel the myth that "something is there for a reason."* |  | ***X*** |  |  |
| *Students commonly misunderstand evolutionary mechanisms but this is not exclusive to Evolutionary Medicine classes. In evolutionary medicine it shows most when talking about antibiotic resistance, however.* |  | ***X*** |  |  |
| *Students mostly struggle with the idea of mutations being the source of evolution: especially when talking about microbial evolution and human evolution.* |  | ***X*** |  |  |
| *Students often lack the preparation in basic evolutionary theory (or worse, have incorrect assumptions about how evolutionary processes work) and in basic human biology/immunology. When they do not full understand the mechanisms they are more likely to make generalizations about the topic and fall in to the trap of the 'just so' story.* | ***X*** | ***X*** |  |  |
| *The biggest one is that evolution has no direction. Many phrase their arguments in teleological fashion.* |  | ***X*** |  |  |
| *Based on the small sample sizes from the first two trial runs of the course, the students in our course are enthusiastic (especially about health-related topics). However, they tend to be weak with regard to quantitative skills and this can cause them difficulty when they try to appreciate the interplay between evolutionary forces.* |  |  |  | ***X*** |
| *The problems that I have mainly come from students who don't have a background in bio anth. The other big problem is culture. They don't know how to talk about it or integrate it with evolutionary topics.* | ***X*** |  |  | ***X*** |
| *The progression of human evolution, specifically the species of humans over time to homo sapiens causes some confusion, as does proximate vs. ultimate causes.* |  | ***X*** |  |  |
| *They struggle with how to untangle their assumptions and those of the researchers whose papers they read from data and analyses* |  |  |  |  |
| *They struggle with the just so stories - adaptationist thinking. But we work hard to address that issue whenever it emerges. Perspectives on phylogenetic shifts often baffle some of the students. I should say that half of the class is "pre-med" with almost no background in evolution but 1/4 are EEB students and 1/4 anth so we work to keep the evolutionary thinking as clear as possible.* | ***X*** | ***X*** |  |  |
| *This is a course designed for non-majors, so there are many things they find challenging. Most notably, the fact that selection can occur on many levels is challenging for them, as is the idea that evolution can be neutral or maladaptive. Understanding the sources of genetic variation is also a major challenge.* | ***X*** | ***X*** |  | ***X*** |
| *Too many to list! My students are in their first semester. Most have good biology backgrounds from high school, but their experience and knowledge are basic. Yet, they are very curious and smart a steep learning curve for everything that they scale quickly.* | ***X*** |  |  | ***X*** |
| *Trade-offs, evolution of virulence (tradeoff hypothesis).* |  | ***X*** |  |  |
| *Varies among students - some have had difficulty with evolutionary psychology ideas regarding sexual reproduction and mate choice. But most have been fine.* |  | ***X*** |  | ***X*** |
| *Virulence-Transmission trade-offs.* |  | ***X*** |  |  |
| *Yes, all of evolutionary biology. Our students are so used to memorizing that any attempt to get them to think conceptually from principles is difficult. I've overcome some barriers (such as how to explain the apparent directionality of random processes in evolution) by using hands-on exercises (we play "evolutionary poker" in which the winning hand is retained -- i.e., reproduces -- while everyone else's hand "dies", which results in ever better hands being selected over many rounds of play -- this is a very successful approach!)* |  | ***X*** |  |  |
| *I cannot think of any.* |  |  | ***X*** |  |
| *I have not noticed any trends.* |  |  | ***X*** |  |
| *It seems to vary every year.* |  |  | ***X*** |  |
| *n/a* |  |  | ***X*** |  |
| *No.* |  |  | ***X*** |  |
| *No.* |  |  | ***X*** |  |
| *No.* |  |  | ***X*** | ***X*** |
| *No, it differed based on the composition of the students in the course.* | ***X*** |  | ***X*** |  |
| *No.* |  |  | ***X*** |  |
| *Not especially. I require a good background in evolution to be able to take the course.* | ***X*** |  | ***X*** |  |
| *Not yet, in my limited experience teaching EM.* |  |  | ***X*** |  |
